# Degeneration-Aware Localization with Arbitrary Global-Local Sensor Fusion

**DOI:** 10.3390/s21124042

**Published:** 2021-06-11

**Authors:** Xiaqing Ding, Fuzhang Han, Tong Yang, Yue Wang, Rong Xiong

**Affiliations:** 1 College of Control Science and Engineering, Zhejiang University, Hangzhou 310027, China; xqding@zju.edu.cn (X.D.); 21960024@zju.edu.cn (F.H.); tong.yang@zju.edu.cn (T.Y.); rxiong@zju.edu.cn (R.X.); 2 State Key Laboratory of Industrial Control and Technology, Hangzhou 310027, China

**Keywords:** multi-sensor fusion, global localization, degeneration-aware state estimation

## Abstract

Global localization is a fundamental ability for mobile robots. Considering the limitation of single type of sensor, fusing measurements from multiple sensors with complementary properties is a valuable task for study. In this paper, we propose a decoupled optimization-based framework for global–local sensor fusion, which fuses the intermittent 3D global positions and high-frequent 6D odometry poses to infer the 6D global localization results in real-time. The fusion process is formulated as estimating the relative transformation between global and local reference coordinates, translational extrinsic calibration, and the scale of the local pose estimator. We validate the full observability of the system under general movements, and further analyze the degenerated movement patterns where some related system state would be unobservable. A degeneration-aware sensor fusion method is designed which detects the degenerated directions before optimization, and adds constraints specifically along these directions to relieve the effect of the noise. The proposed degeneration-aware global–local sensor fusion method is validated in both simulation and real-world datasets with different sensor configurations, and shows its effectiveness in terms of accuracy and robustness compared with other decoupled sensor fusion methods for global localization.

## 1. Introduction

Precise global localization is of high importance in many applications, such as navigation and mapping. When there is no access to a pre-built environment map, the robot can only obtain the global positioning information via global-aware sensors like global positioning system (GPS) and ultra wide band (UWB). Generally these sensors can access global positional information with bounded error. However, due to the installation limitation of the ground stations and the signal block from obstacles, their measurements might be intermittent and suffer from large noise. Besides, the 3 degree-of-freedom (3D) positioning measurements are inadequate for some applications that require 6 degree-of-freedom (6D) global pose estimation. On the other hand, local sensors that measure the local environmental or kinematic information can achieve consecutive 6D pose estimation in real time [1,2,3,4,5]. However, the poses are estimated relative to the origins of the trajectories, which is unobservable in the global reference frame. The estimation results would drift along with time and distance.

In this paper, we set to integrate the global and local sensors to achieve 6D global drift-free localization. To do this, the extrinsic calibration between sensors is required, and so is the mapping that transforms the local estimations to the global coordinate. Besides, the scale of the local poses is also required since some local estimators such as monocular visual odometry can not recover it. Many researches investigate the sensor-fusion problem on specific sensor configurations, and the methods can be divided into two categories.

One kind of fusing methods tightly couple the measurements from different sensors under the filtering or optimization based frameworks [6,7]. Generally, the reference frames of the local estimators are directly transformed to the global coordinates after initialization. During localization, the poses of the local estimators are propagated on the global coordinate. These methods are usually carefully designed for specific sensor configurations, and cannot be generalized to arbitrary configuration. To the best of our knowledge, none of these methods are compatible with the local estimators that cannot recover the absolute scale. Another kind of methods utilize the outputs from different pose estimation systems as observation, and loosely couple them to achieve global localization [2,8,9]. In this way, the pose estimation systems are decoupled, and the erroneous estimations or measurements from one system would not affect the others. Besides, the loosely-coupled methods are applicable to arbitrary sensor configurations due to the independency of the pose estimation front-ends [8,9,10].

However, few of these methods have analyzed the observability of the global–local sensor fusion system. As noted in [6], the sensor fusion systems would still remain globally unobservable even if the global measurements are given. Besides, the observability of the system would degenerate when the robot moves along certain motion patterns [11,12]. Many studies analyze the observability to guide the online movements or calibration design [11,13], but few of them pays attention to avoiding degeneration from worsening localization performance. One possible solution to address degeneration is to add heuristic constraints on the system, e.g., priors like constant velocity or planar motion models, which however would introduce false constraints on observable subspace if the hypothesis is violated. Some works [14,15] address the degeneration occurred in laser odometry and calibration, respectively. They first determine the unobservable directions according to the eigenvalues of systems, and truncate the state update along with these directions. These methods theoretically prevent noise from deteriorating unobservable states, but the eigenvalue threshold for observability distinction might change from case to case, and the optimizer has to be adapted for the truncated update.

In this paper, we propose a novel global–local sensor fusion framework that estimates the translational extrinsic calibration, the scale of the local poses, and the relative transformation from the reference frame of the local estimator to the global coordinate within a sliding-window, which are utilized to map the real-time local poses to the global frame for localization. We prove the full observability of this formulation under general motion patterns. To avoid the degeneration from deteriorating state estimation, we distinguish the degenerated subspace before each optimization, and add prior constraints specifically along with the unobservable directions. We validate the effectiveness of the proposed method in both simulated and real-world datasets with different sensor configurations. The experimental results also show that our method can effectively restrain the drift along degenerated directions and work out robust global localization results. To summarize, the contributions of the paper are listed as follow

- A novel global-local sensor fusion framework is proposed which is applicable to arbitrary sensor configurations. The observability and degenerated motion patterns of the proposed system are detailedly analyzed;
- A degeneration-aware optimization approach is designed to avoid the sensor fusion framework being deteriorated by the noise on the degenerated directions;
- Both simulated and real-world datasets with multiple sensor configurations are utilized to validate the generalization and effectiveness of the degeneration-aware sensor fusion framework for global localization.

## 2. Related Works

The study in this paper is relative to multi-sensor fusion and degeneration-aware state estimation. Considering the limitation of the single type of sensor, multi-sensor fusion is a widely researched area in robotics. Generally, the sensors selected for fusion possess complementary properties. For example, the monocular visual inertial system fuses external visual information with internal inertial information to estimate 6D pose with scale. In this paper, we pay attention to the fusion between local-aware sensors (e.g., light detection and ranging (LIDAR), camera, inertial measurement unit (IMU)), and global-aware sensors (e.g., GPS, Motion Capture) for localization. The fusion frameworks can be divided into filtering and optimization-based methods. Typically, most of the filtering-based frameworks are developed based on Extended Kalman Filter (EKF) [6,8,16] and utilize global and local information to update the propagated inertial data. To reduce the first-order linearization error occurred in the EKF, some systems achieve sensor fusion taking advantages of the other filtering solutions such Partical Filter [17,18] or Sigma-point Kalman Filter [19,20]. Lynen et al. [8] propose a general multi-sensor fusion (MSF) method that can utilize arbitrary global or local measurements to update the propagated pose, in which the scale of the local estimator and the extrinsic calibration could also be estimated during the localization. However, the inertial instrument is indispensable and the observability is not validated in this method. Lee et al. [6] tightly couple the visual, inertial, and GPS information for global localization, and simultaneously estimates the extrinsic calibration and time-offset between sensors. Optimization-based methods are proven to be more accurate than filtering-based methods [21]. During optimization, the system states are iteratively estimated based on all of the valid measurements, which however require a large number of computational resources. Many works relieve the computational burden by only maintaining a sliding-window of states to achieve efficient optimization [9,22,23]. However, few of these fusion methods address the degeneration problem during online localization.

Observability is the vital property of dynamic systems which reflects whether the inner states of the systems can be estimated from the observed measurements [24]. In the global–local sensor fusion tasks, Lee et al. [6] prove that if directly adding the relative transformation between the reference frames of the visual inertial odometry (VIO) and GPS systems into the original VIO state for estimation, there still exists four unobservable directions even though the global measurements exist. They address this problem by transforming the states of the local VIO system to the global frame to achieve full observability, which is inherently equivalent to marginalizing out the relative transformation between the reference frames of VIO and GPS systems after initialization, and directly estimating the state of the local VIO system in the global frame. This parameterization is also utilized in some of the other global–local sensor fusion methods [7,10]. Another approach decouples the local and global estimators and estimates the relative transformation between their reference frames [25]. In this way the output of the high-frequency local estimator can be directly transformed to the global frame without latency, and makes the system more robust as the local and global estimators are decoupled. In this paper, besides the relative transformation between reference frames, we further add the scale of the local estimator and the extrinsic calibration between local and global sensors into the state, which makes the system applicable to arbitrary sensor configurations. We also prove its full observability under general movements.

Even though the fusion system is proved to be fully observable in general conditions, the observability would degenerate if the robot moves with insufficiently excited motion. Ref. [11] proves in detail that there exist several motion patterns that would introduce unobservable directions even if the global measurement is provided in the inertial navigation system (INS) aided framework. However, few works propose efficient solutions towards preventing degeneration from deteriorating estimation results. Refs. [14,15] address the degeneration problem by projecting the incremental update to the observable direction, which needs to modify the update step in general optimizers. In this paper, we automatically detect the degenerated directions before optimization, and specifically add constraints based on the calculated directions to prevent the unobservable parameters from being deteriorated by noise, which can be easily plugged in many existing systems developed with general optimizers.

## 3. Method

In this section, first the notations used throughout the paper are presented. Then we introduce the formulation of the global–local sensor fusion system and its detailed implementation for batch optimization. As a special discussion, we show that some commonly-appeared motions in robotics are actually degenerated for sensor fusion. Finally, we present our degeneration-aware optimization method that is designed to address the degeneration problem occurred during online localization.

### 3.1. Notation

**Figure 1:**
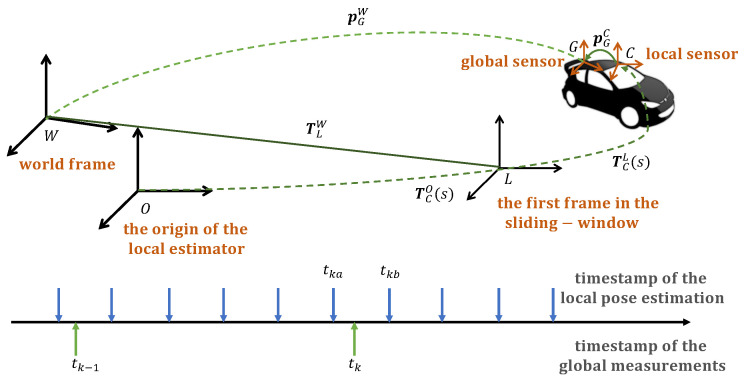
The demonstration of the frames and timeline utilized throughout the paper.

The nomenclature used throughout the paper is defined in Table 1. We consider that the local estimator outputs 6D transformation $T_{C}^{O}$ from the local sensor frame *C* to the reference frame of the odometry *O*. We assume that the global estimator outputs 3D positional measurement $p_{G}^{W}$ of the global sensor frame *G* on the robot with respect to a fixed world frame *W*. To fuse the outputs from the global and local estimators, we estimate the extrinsic calibration $T_{G}^{C}$ between sensors, the scale *s* of the local estimator, and the relative transformation $T_{O}^{W}$ during online localization, which constitute the system state x. T(s) denotes that the transformation T is mapped to the real scale by *s*. We draw the relation of the frames in Figure 1.

### 3.2. Global–Local Sensor Fusion System

**Figure 2:**
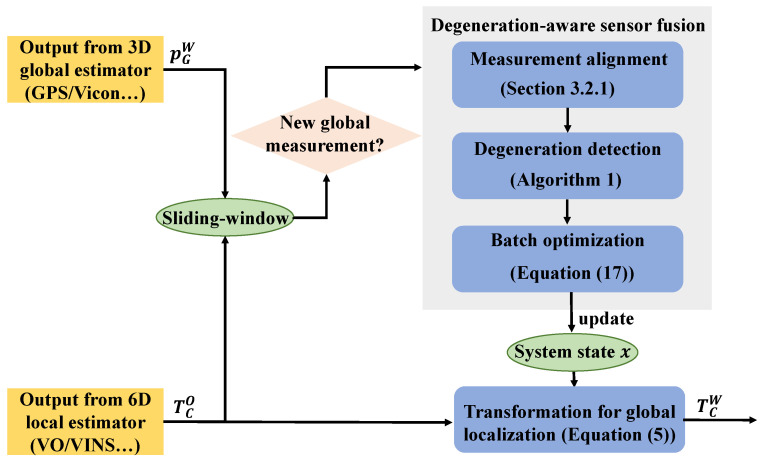
The pipeline of the proposed degeneration-aware sensor fusion framework.

The proposed sensor fusion system is demonstrated in Figure 2. It receives outputs from arbitrary local and global estimators, and produces the global localization results at the highest frequency of the local estimator. We estimate the transformation $T_{O}^{W}$ to transform the output of the local estimator to the global frame instead of directly estimating the current pose in the global frame. To restrict the computational requirement and estimate the scale accurately, the fusion process is carried out on a sliding-window of recent *n* global sensor measurements and the related local sensor frames within the corresponding time duration, and we denote the first local sensor frame in the sliding-window as *L*. The length of the sliding-window in this work is maintained according to the distance, but can be modified by any other criterion. Upon the sliding-window based formulation, we change the estimated relative transformation from $T_{O}^{W}$ to $T_{L}^{W}$ and the system state is maintained accordingly as

$$x=\{R_{L}^{W},p_{L}^{W},p_{G}^{C},s\}$$

Generally, the global sensors possess lower frequency than the local estimators. Therefore, the system performs the fusion process each time it receives the measurement from the global estimator. As shown in Figure 2, during each fusion process, we first align the newly received global measurement with the two closest local frames to obtain the accurate local pose at the exact timestamp of the global measurement. Then, we detect whether there exists degenerated subspace according to the information within the sliding-window. If the degeneration occurs, the corresponding degenerated directions would be calculated, which further are utilized to construct prior constraints for degeneration-aware optimization.

#### 3.2.1. Measurement Alignment

We first align the global and local measurements according to the timestamps. The global measurement $p_{G_{k}}^{W}$ received at timestamp $t_{k}$ is aligned with two closest local measurements at timestamp $t_{ka}$ and $t_{kb}$ as shown in Figure 1. We interpolate the corresponding local pose $T_{C_{k}}^{O}$ at the timestamp $t_{k}$ according to

(1) $$\begin{array}{cc} \; & \lambda =\frac{t_{k}-t_{ka}}{t_{kb}-t_{ka}}\\ q_{C_{k}}^{O}= & \mathrm{slerp}(q_{C_{ka}}^{O},q_{C_{kb}}^{O},\lambda )\\ p_{C_{k}}^{O}= & \left(1-\lambda \right)p_{C_{ka}}^{O}+\lambda p_{C_{kb}}^{O} \end{array}$$

in which slerp(·) denotes the spherical linear interpolation function [26].

#### 3.2.2. General Batch Optimization for Localization

After aligning the newly received global measurement, the system state $x=\{R_{L}^{W},p_{L}^{W},p_{G}^{C},s\}$ is optimized according to the time-aligned measurements within the current sliding-window. The error function constructed by the measurements at timestamp $t_{k}$ is formulated as

(2) $$e_{p_{k}}={\bar{p}}_{G_{k}}^{W}-p_{G_{k}}^{W}$$

in which ${\bar{p}}_{G_{k}}^{W}$ is derived by

(3) $$\begin{array}{cc} T_{C_{k}}^{L}= & \left[\begin{array}{cc} R_{C_{k}}^{L} & p_{C_{k}}^{L}\\ 0_{1\times 3} & 1 \end{array}\right]={\left(T_{L}^{O}\right)}^{-1}T_{C_{k}}^{O}\\ T_{G_{k}}^{W}= & \left[\begin{array}{cc} R_{G_{k}}^{W} & p_{G_{k}}^{W}\\ 0_{1\times 3} & 1 \end{array}\right]=T_{L}^{W}T_{C_{k}}^{L}\left(s\right)T_{G}^{C}\\ = & \left[\begin{array}{cc} R_{L}^{W} & p_{L}^{W}\\ 0_{1\times 3} & 1 \end{array}\right]\left[\begin{array}{cc} R_{C_{k}}^{L} & sp_{C_{k}}^{L}\\ 0_{1\times 3} & 1 \end{array}\right]\left[\begin{array}{cc} R_{G}^{C} & p_{G}^{C}\\ 0_{1\times 3} & 1 \end{array}\right]\\ {\bar{p}}_{G_{k}}^{W}= & R_{L}^{W}R_{C_{k}}^{L}p_{G}^{C}+sR_{L}^{W}p_{C_{k}}^{L}+p_{L}^{W}\\ = & R_{L}^{W}\left(R_{C_{k}}^{L}p_{G}^{C}+sp_{C_{k}}^{L}\right)+p_{L}^{W} \end{array}$$

Summing up all the valid error functions in the sliding-window, the cost function for the general localization based optimization is defined as

(4) $$f=\sum_{k}\rho (e_{p_{k}}^{T}\Omega_{e}e_{p_{k}})$$

in which $\Omega_{e}$ denotes the information matrix that can be derived as the inverse of the measurement covariance matrix.ρ(·) represents the Huber’s robust kernel function [27] which can suppress the effect of outliers. In this work, we minimize this non-linear least-square cost function iteratively for system state optimization based on the Levenberg-Marquardt algorithm [28,29].

Given the latest optimized state x, we can transform the continuous output of the local estimator $T_{C_{t}}^{L}$ to the world frame for real-time 6D global localization. The 6D global localization at timestamp *t* can be calculated as

(5) $$T_{C_{t}}^{W}=\left[\begin{array}{cc} R_{L}^{W}R_{C_{t}}^{L} & sR_{L}^{W}p_{C_{t}}^{L}+p_{L}^{W}\\ 0_{1\times 3} & 1 \end{array}\right]$$

### 3.3. Observability Analysis

For the optimization formulated in Equation (4), we first analyze its observability in the general occasion. We linearize Equation (2) at the current estimate to compute the Jacobian

(6) $$H_{k}=\frac{\partial e_{p_{k}}}{\partial x}={\left[\begin{array}{cccc} \Xi_{k} & I_{3\times 3} & R_{L}^{W}R_{C_{k}}^{L} & R_{L}^{W}p_{C_{k}}^{L} \end{array}\right]}_{3\times 10}$$

in which $\Xi_{k}$ corresponds to the Jacobian of $R_{L}^{W}$ on Manifold

(7) $$\Xi_{k}=-R_{L}^{W}\left[\left(R_{C_{k}}^{L}p_{G}^{C}+sp_{C_{k}}^{L}\right)\times \right]$$

We denote [a×] as the 3×3 skew-symmetric for vector $a={\left[a_{x},a_{y},a_{z}\right]}^{T}$, which is formulated as

(8) $$\begin{array}{c} \left[a\times \right]=\left[\begin{array}{ccc} 0 & -a_{z} & a_{y}\\ a_{z} & 0 & -a_{x}\\ -a_{y} & a_{x} & 0 \end{array}\right] \end{array}$$

As the system state x changes slowly across time, the corresponding state transition matrix $\Phi (t_{0},t_{k})$ between timestamps $t_{0}$ and $t_{k}$ is identity. The observability matrix M can be constructed following [30]

(9) $$M=\left[\begin{array}{c} \vdots \\ H_{k}\Phi \left(t_{0},t_{k}\right)\\ \vdots \end{array}\right]=\left[\begin{array}{c} \vdots \\ H_{k}\\ \vdots \end{array}\right]$$

As in general cases the columns in $H_{k}$ are linear independent. Thus based on Equation (9) it can be noticed that in general cases the rank of M achieves 10 when n≥4. Therefore, when there are more than 4 global measurements in the sliding-window, the system is observable.

### 3.4. Degeneration Analysis

In this subsection, we summarize the common degenerated motion patterns in the robotics area and derive their corresponding unobservable directions.

#### 3.4.1. Pure Translation along One Axis

This is a common motion pattern in autonomous driving. When the robot moves along a straight line, $R_{C_{i}}^{L}$ is invariant for any $i\in \;\left[m,m+n-1\right]$, in which *m* is utilized to denote the beginning of the sliding-window. The translational measurement of the local estimator can be derived as

(10) $$p_{C_{i}}^{L}=\alpha_{i}{\bar{p}}_{m_{0}}^{L}$$

in which ${\bar{p}}_{m_{0}}^{L}$ is the unit vector of the translational direction and $\alpha_{i}$ is a scalar variable that can be derived as $\alpha_{i}=\;∥p_{C_{i}}^{L}∥/∥{\bar{p}}_{m_{0}}^{L}∥$. Thus, in $H_{i}$ the third block is constant, and the first two blocks are also linear dependent. The right null-space matrix N of the observability matrix M can be derived as

(11) $$N={\left[\begin{array}{cc} 0_{3\times 3} & {\bar{p}}_{m_{0}}^{L}\\ -R_{L}^{W}R_{C_{i}}^{L} & R_{L}^{W}\left[R_{C_{i}}^{L}p_{G}^{C}\times \right]{\bar{p}}_{m_{0}}^{L}\\ I_{3\times 3} & 0_{3\times 1}\\ 0 & 0 \end{array}\right]}_{10\times 4}$$

MN=0 can be verified as $H_{i}N=0$. Therefore, in this occasion, there is a four-dimensional unobservable subspace which is constructed as span(N). The formation of N shows that the first three column vectors are related to the translational components $\left\{p_{L}^{W},p_{G}^{C}\right\}$, while the last column vector is related to one direction of $R_{L}^{W}$ which is parallel to the translational direction ${\bar{p}}_{m_{0}}^{L}$.

#### 3.4.2. Random 3D Translation

This would occur when the robot performs pure translation in 2D/3D space without rotating. In this case, Equation (10) is violated while $R_{C_{i}}^{L}$ keeps invariant, which decreases the dimension of N to 3

(12) $$N={\left[\begin{array}{c} 0_{3\times 3}\\ -R_{L}^{W}R_{C_{i}}^{L}\\ I_{3\times 3}\\ 0 \end{array}\right]}_{10\times 3}$$

We can find that $R_{L}^{W}$ is observable now, and there remain three unobservable directions that are related to the translational components $\left\{p_{L}^{W},p_{G}^{C}\right\}$.

#### 3.4.3. One-Axis Rotation

When the robot turns along a fixed rotation axis $\omega \in R^{3}$ with non-zero translational velocity, only the translational components along ω remain unobservable. In this case, $R_{C_{i}}^{L}$ can be derived according to the Rodrigues’ rotation formula

(13) $$R_{C_{i}}^{L}=R_{C_{m}}^{L}\left(I_{3\times 3}+\sin\left(\theta_{i}\right)\left[\omega \times \right]\;+\;\left(1-\cos\left(\theta_{i}\right)\right){\left[\omega \times \right]}^{2}\right)$$

in which $\theta_{i}$ denotes the rotation angle of $R_{C_{i}}^{C_{m}}$. As $\left[\omega \times \right]\omega =0_{3\times 1}$, we can derive that multiplying the third block of $H_{i}$ by ω leads to

(14) $$R_{L}^{W}R_{C_{i}}^{L}\omega =R_{L}^{W}R_{C_{m}}^{L}\left(I_{3\times 3}+\sin\left(\theta_{i}\right)\left[\omega \times \right]+\left(1-\cos\left(\theta_{i}\right)\right){\left[\omega \times \right]}^{2}\right)\omega =R_{L}^{W}R_{C_{m}}^{L}\omega$$

The result is constant for any $i\in \;\left[m,m+n-1\right]$. Thus, the null-space matrix that satisfies MN=0 can be derived as

(15) $$N={\left[\begin{array}{cccc} 0_{1\times 3} & -{\left(R_{L}^{W}R_{C_{m}}^{L}\omega \right)}^{T} & \omega^{T} & 0 \end{array}\right]}_{10\times 1}^{T}$$

It should be noticed that when the robot performs uniform motion, more dimensions maybe unobservable. One case is that, when both the angular velocity and the translational velocity are constant, the dimension of the unobservable subspace is expanded to 2 as one direction of $R_{L}^{W}$ that is along with the rotational axis would be undistinguishable. Another example is that, when the directions of the constant angular and translational velocity are perpendicular, the scale of the local estimator would be unobservable. These are likely to happen when the robot performs planar rotational movement, such as a car turns with constant velocity in autonomous driving. Detailed proof of the two claims are derived in the Appendix A.

### 3.5. Degeneration-Aware Batch Optimization

When there exists an unobservable subspace in the system, the related states are unconstrained and susceptible to measurement noise. To address this problem, we first detect the degenerated directions before the optimization process, then add prior constraints specifically along these directions.

Similarly to [14], we calculate the eigenvalues $\left\{\lambda_{l}\right\}$ and the corresponding eigenvectors $\left\{v_{l}\right\}$ by performing SVD decomposition on the Hessian matrix $H^{T}H$, in which $H={\left[H_{m}^{T}\cdots H_{m+n-1}^{T}\right]}^{T}$ is linearized at the latest estimated state before each optimization and l=0⋯9. We assume that $\left\{\lambda_{l}\right\}$ is sorted and $\lambda_{9}$ corresponds to the smallest eigenvalue. Theoretically the eigenvalues corresponding to the unobservable dimensions are precisely zero, while in practice they are not because of the system noise. It is apparent that a constant threshold ϵ is not sufficient to distinguish zero eigenvalues for all kinds of systems, calling for more flexible construction of the threshold. We also quantitatively analyze the influence of noise on eigenvalues in Section 4.1.

We propose a heuristic method to detect the unobservable subspace as shown in Algorithm 1. The absolute thresholds $\epsilon_{a}$ and $\epsilon_{b}$ define the coarse but valid upper and lower bounds distinguishing the unobservable and observable subspaces. For the eigenvalue that distributes between the two bounds, it is difficult to directly categorize it by a threshold due to the unrestricted range of noise. We compute its ratio with the adjacent eigenvalues to indicate whether they have the same type of observability. If the ratio is larger than threshold $\epsilon_{r}$, we assume that the adjacent eigenvalues have the same type of observability. As the method traverses from the smallest eigenvalue, the eigenvalue is categorized as relating to the unobservable subspace if it shares the same type of observability with its previous one.
**Algorithm 1:** Detecting unobservable directions.
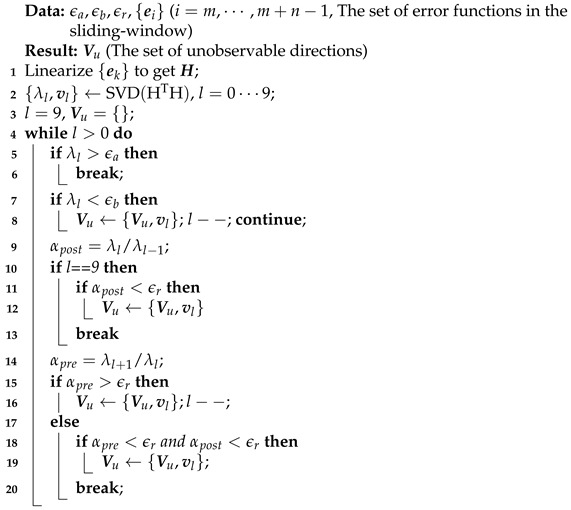


After detecting out the degenerated subspace with dimension *r*, we collect the corresponding eigenvectors $V_{u}=\left\{v_{0},\cdots ,v_{r-1}\right\}$ which denote the degenerated directions. Instead of projecting the incremental update to the observable directions during optimization, we restrict the update in unobservable directions by adding constraints

(16) $$\begin{array}{cc} e_{R_{j}} & =\log\left(({\hat{R}}_{L}^{W}{)}^{T}R_{L}^{W}\right)v_{j(0:3)}\\ e_{p_{Lj}^{W}} & =({\hat{R}}_{L}^{W}{)}^{T}\left(p_{L}^{W}-{\hat{p}}_{L}^{W}\right)v_{j(3:6)}\\ e_{e_{j}} & =\left({\hat{p}}_{G}^{C}-p_{G}^{C}\right)v_{j(6:9)}\\ e_{s_{j}} & =\left(\hat{s}-s\right)v_{j(9)} \end{array}$$

in which j∈[0,r−1]. $e_{R_{j}},e_{p_{Lj}^{W}}$, $e_{e_{j}}$ and $e_{s_{j}}$ denote the constraints applied on $R_{L}^{W},p_{L}^{W}$, $p_{G}^{C}$ and *s*, respectively. $\left(\hat{\cdot }\right)$ denotes the related state estimation before optimization. $v_{j(a:b)}$ represents the vector that is compromised with the *a*th to *b*th elements of $v_{j}$. These error terms can be considered as priors to prevent the states from drifting in the unobservable subspace, and are only added for optimization when $∥v_{j(a:b)}∥\;>0.1$, which means that the prior constraint on this state is activated. Finally the cost function in the proposed degeneration-aware batch optimization is

(17) $$\begin{array}{cc} f= & \sum_{i=m}^{m+n-1}\rho (e_{p_{i}}^{T}\Omega_{e}e_{p_{i}})+\sum_{j\in S_{R}}e_{R_{j}}^{T}\Omega_{p}e_{R_{j}}+\sum_{j\in S_{p_{L}^{W}}}e_{p_{Lj}^{W}}^{T}\Omega_{p}e_{p_{Lj}^{W}}\\ \; & +\sum_{j\in S_{e}}e_{e_{j}}^{T}\Omega_{p}e_{e_{j}}+\sum_{j\in S_{s}}e_{s_{j}}\Omega_{ps}e_{s_{j}} \end{array}$$

in which $S_{R},S_{p_{L}^{W}},S_{e}$ and $S_{s}$ denote the sets of activated prior constraints on the system states, respectively. $\Omega_{p}$ and $\Omega_{ps}$ are the information matrices of the prior constraints.

## 4. Experimental Results

We validate the effectiveness of our degeneration-aware sensor fusion method through both simulated and real-world datasets. In simulation experiments, we first show the sensitivity of the eigenvalues with respect to the measurement noise. Then we create four sequences of simulated trajectories that correspond to the common degenerated motion patterns analyzed in Section 3.4, and utilize them to demonstrate the effect of degeneration on state estimation and the effectiveness of our proposed method in terms of preventing the state from drifting along the degenerated directions. In real-world experiments, we show the localization performance on indoor EuRoC [31] and outdoor KAIST [32] datasets. EuRoC is a visual-inertial dataset collected by a micro aerial vehicle that performs 6D movements. We utilize the 3D Leica MS50 laser tracker as the 3D global estimator and only utilize the left images to perform monocular visual odometry, which serves as the local pose estimator with no access to the absolute scale. KAIST dataset is collected in outdoor urban environment on a car and we utilize the GPS as global estimator and stereo-IMU information to perform local visual-inertial odometry. During the experiments, we set the length of the sliding-window as 10 m for indoor dataset and 50 m for outdoor datasets. All the experiments are executed on a laptop with a 2.70 GHz Intel Core i7-7500U CPU. The codes are implemented in C++ and the optimization process is implemented with Levenberg-Marquardt algorithms in g2o [33].

In the simulation experiments, different motion patterns are controlled by setting different translational and rotational velocities for the local sensor. The minimum sampling interval is 0.1 s, and the ground truth of the trajectories can be integrated based on the velocities. The trajectories of the global sensor can be calculated based on the ground truth of the system state using Equation (3). The global measurements are simulated by corrupted the ground truth of the trajectories with Gaussian noise. To simulate the drift of the local estimator, we add Gaussian noise on the relative pose in each sampling interval, and integrate the corrupted relative pose to formulate the local measurements. As the ground truth of the system state can be accessed in the simulation experiments, the performances of different methods are evaluated by comparing the state estimation errors.

In real-world experiments, we evaluate the performances of different methods by the absolute trajectory error (ATE) and rotational error [34]. For each global pose $T_{C_{t}}^{W}$ estimated at timestamp *t*, we find its corresponding ground truth pose $T_{C_{t}}^{W*}$ provided by the datasets according to the timestamp. The localization error is derived as

(18) $$E_{t}={\left(T_{C_{t}}^{W*}\right)}^{-1}T_{C_{t}}^{W}$$

The ATE error relates to the norm of the translational component in $E_{t}$, and the rotational error relates to the rotational component in $E_{t}$.

### 4.1. Sensitivity of the Eigenvalues

**Figure 3:**
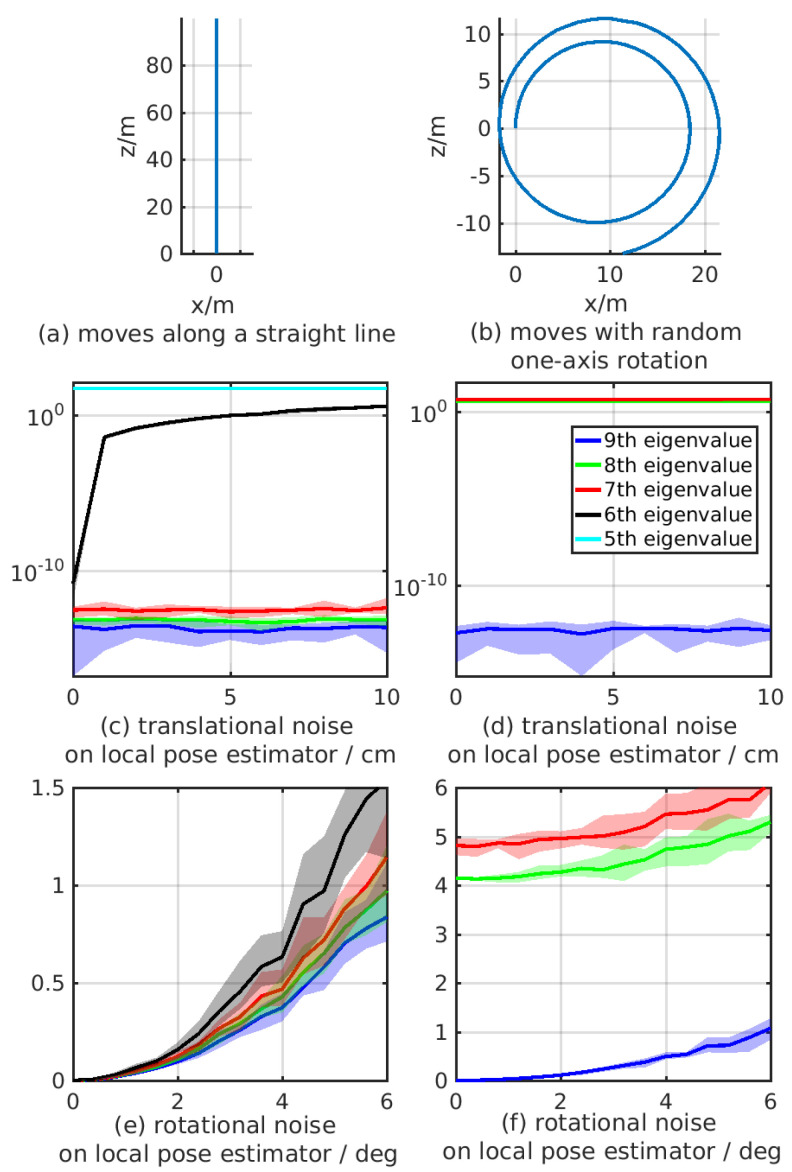
Eigenvalues of the system calculated on two degenerated trajectories with different measurement noises. The curves in each figures denote the means of the eigenvalues and the colored region surrounding the curve represents the statistic of 200 trials.

To quantitatively demonstrate the influence of the noise on the eigenvalues, we create simulated trajectories that are related to two types of the degenerated motion patterns shown on the first row of Figure 3. We add Gaussian noise on the simulated measurement and show the changing of the related eigenvalues. As the derivation of eigenvalues relates to the measurements of the local estimator, we, respectively, add Gaussian noises with standard deviations from 0 to 10 cm on the translational parts of the local measurements, and add Gaussian noises with standard deviations from 0 to 6 deg on the rotational parts. To avoid the influence of randomness, for each standard deviation we generate 200 trajectories for eigenvalue evaluation. Figure 3a shows the trajectory generated when the robot moves along a straight line and the eigenvalues drawn in Figure 3c,e are influenced by translational and rotational noises on the local estimators with different standard deviations, respectively. To keep the scale of the axis for better demonstration, we do not draw the value of the 5th eigenvalue influenced by the rotational noise in Figure 3e, which are around the same values as shown in the results that influenced by the translational noise. The right column includes the results tested when the robot moves with random one-axis rotation. The trajectory is shown in Figure 3b. The eigenvalues drawn in Figure 3d,f are influenced by the translational and rotational Gaussian noises, respectively. The curves in each figures denote the means of the eigenvalues and the colored region surrounding the curve represents the statistic of 200 trials. Some colored regions are hard to be distinguished as they are too close to the curve in the related axis scale.

Based on our analysis in Section 3.4, when the robot moves along a straight line, there should be four unobservable directions, which means that the smallest four eigenvalues should be close to zero. When the robot moves with non-constant one-axis rotation, there should be only one unobservable direction. We, respectively, draw the smallest five and three system eigenvalues tested on the two trajectories in the two columns of Figure 3. The results show that when the noise is small, the eigenvalues corresponding to the unobservable subspace are close to zeros (i.e., the 6–9th eigenvalues on the first column and the 9th eigenvalue on the second column), and become larger when the noise grows. Although the eigenvalues corresponding to the observable subspace are generally large. Thus we could set the coarse upper bound $\epsilon_{a}$ and lower bound $\epsilon_{b}$ to distinguish eigenvalues. However, we can notice that the 6th eigenvalue on the left column is close to the 7th and 8th eigenvalues on the right column. If we utilize one constant threshold, it is difficult to distinguish the unobservable and observable subspaces in this situation. In our proposed detection method, the difference between the adjacent eigenvalues is taken into consideration, which contributes to handle this problem. Based on the analysis of these results, we set $\epsilon_{a}=5,\epsilon_{b}=10^{-2},\epsilon_{r}=0.1$ throughout the left of the experiments.

### 4.2. Simulation Results

In this subsection, we utilize simulated data collected under degenerated movement patterns to perform ablation study on the effectiveness of the proposed degeneration-aware sensor fusion. Specifically, we evaluate the accuracy of the estimated system state optimized with general optimization formulated in Equation (4) and the optimization with the degeneration-aware constraints (DC) in Equation (17), respectively. The simulated data includes four types of trajectories in which the robot moves (a) along a straight line, (b) with random 3D translation, (c) with perpendicular constant rotational and translational velocity, and (d) with random one-axis rotation. The motion patterns are controlled by the translational and rotational velocities. We set the sampling time as 0.1 s and the trajectories are integrated based on the velocity. We add zero-mean Gaussian noise on the relative pose within each sampling time to simulate the measurement noise of the local estimator and integrate the relative poses to simulate the local measurements. The 3D global measurements are collected every 1 s along the ground truth of each trajectory and are also corrupted by the noise. The standard deviations of the rotational and translational noises for the local estimator are 0.05 rad and 0.05 m. The standard deviation of the translational noise for the global estimator is 0.5 m.

**Figure 4:**
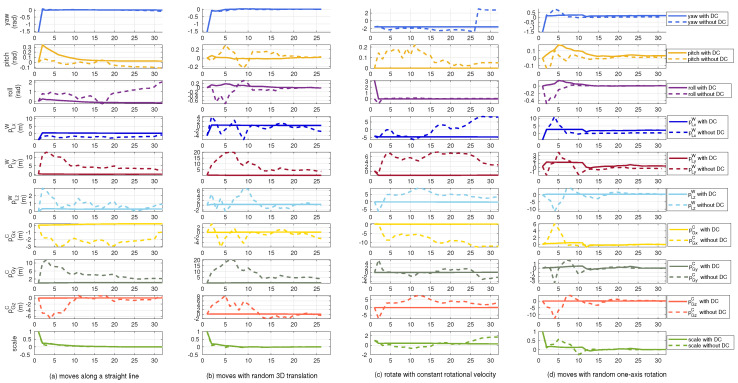
The evaluated state estimation errors tested on the simulation dataset. From top to the bottom the figures in each row present the estimation errors on each element of the system state x. The x-axis denotes the time line with the unit of second.

The estimation errors for the system state x are drawn in Figure 4 and we represent the rotational error of $R_{L}^{W}$ in Euler Angle parameterization denoting as “yaw”, “pitch”, and “roll” in the first three rows of the figure. Based on Equation (11), when the robot moves along a straight line, the dimension of the unobservable subspace is four, which relates to one rotational axis of $R_{L}^{W}$ along the moving direction and 3D translational directions that are coupled in $p_{L}^{W}$ and $p_{G}^{C}$. These can be reflected from the results in the first column of Figure 4 indicated by dashed lines which do not converge in the related dimensions. On the other hand, the results optimized with the proposed degeneration-aware constraints, though could not converge to the ground truth in the unobservable directions as no valid information is provided, do not diverge during the whole process. The results in the second to fourth columns also validate our analysis in Section 3.4. Additionally, it is interesting to notice that, as one direction of the unobservable space might be related to several state variables, *r* dimensions of unobservable directions would cause the divergence of more than *r* state variables.

### 4.3. Real World Experiments

We evaluate the global localization performance of our proposed method on sequences MH01–MH05 in EuRoC datasets and sequence Urban 38 in KAIST dataset.We implement the monocular visual odometry based on ORB-SLAM [1] and the stereo visual inertial odometry based on Openvins [3]. We utilize the absolute trajectory error (ATE) [34] to evaluate the global localization performance and compare our results with the open-sourced multi-sensor fusion methods MSF [8] and VINS-Fusion [10].

#### 4.3.1. EuRoC Dataset

In EuRoC dataset we utilize monocular vision to perform local pose estimation. The output of the monocular visual odometry does not include the absolute scale, which can not be utilized for global localization in many other global–local fusion methods [7,9,10]. Thus, to test the performance of VINS-Fusion [10], we change its local estimator into VINS-MONO [2], which is the classical monocular VIO provided by the VINS-Fusion system.

**Figure 5:**
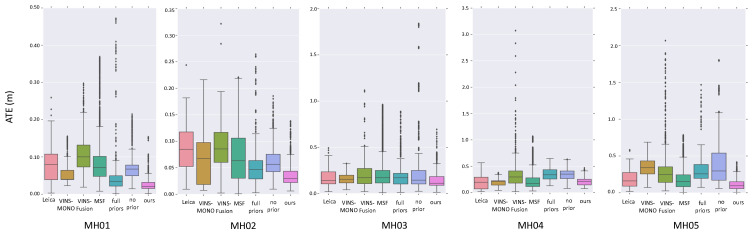
The average trajectory errors of different localizers tested on EuRoC dataset.

We draw ATE errors evaluated by different methods in Figure 5, and list the rotational errors in Table 2. The results of VINS-MONO is evaluated by aligning the trajectories to the ground truth. The results show that our method could successfully fuse 3D global positions and 6D scaleless local poses for global localization, and outperform the compared methods in terms of both translational and rotational localization accuracy. We can notice that in many sequences VINS-MONO also performances well as the trajectories are not long, but the fusion results in VINS-Fusion show larger errors as the extrinsic parameter is not estimated in VINS-Fusion. Besides, compared with MSF and VINS-Fusion, our method does not require IMU data to support sensor fusion, which further demonstrates its efficiency and versatility for sensor fusion based global localization.

We also perform ablation study to evaluate the effectiveness of the degeneration-aware optimization. We first evaluate the localization performance tested on the general batch optimization formulated in Equation (4). The results are denoted as “no prior” in Figure 5. Besides, to validate the benefit of adding prior constraints specifically on the degenerated directions, we also test the localization performance by adding the full prior constraints for the whole system state. The results are denoted as “full priors” in Figure 5. From the results we can infer that, the degeneration constraints can successfully improve the localization accuracy as the influence from the noise in degenerated directions is restrained. Besides, adding constraints especially on degenerated directions can also improve the localization accuracy as the observable elements in the system state could be optimized directly without influenced by heuristic constraints which might be inaccurate.

#### 4.3.2. KAIST Dataset

In KAIST dataset, we utilize the GPS as the global estimator, which is prone to drift when the car stops at the crossroads. Additionally, the GPS signal is inaccurate when the covariance is large. Therefore, during online localization, we drop the GPS messages with large covariance (the threshold is set as 60) and the drifted messages indicating that the car is moving while the local estimator denoting that the car does not move.

**Figure 6:**
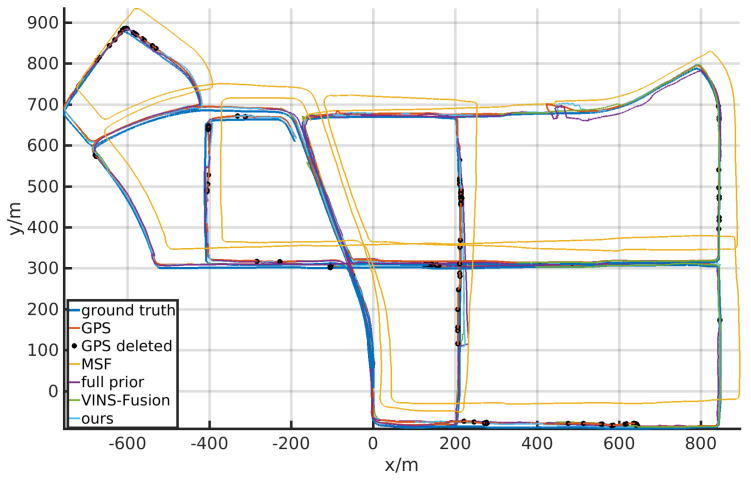
The localization trajectories of different localizers tested on Urban 38 of the KAIST dataset.

We list the localization performance of GPS and the stereo-IMU version of Openvins [3] in Table 3 as baseline, and also compare our results with MSF [8], VINS-Fusion [10], and full priors aided results. The translational and heading errors are listed in Table 3 and Table 4. The corresponding trajectories are drawn in Figure 6. The dropped GPS signals are also marked in the trajectory.

Compared with EuRoC datasets, the robot performs planar movements in Kaist dataset, in which the degeneration would likely occur. Thus, the results of “no prior” are largely influenced by the degeneration and fail to perform the whole localization. The results also show that our method achieves better performance than the compared methods and could provide drift-free global localization results.

### 4.4. Computational Cost Evaluation

**Figure 7:**
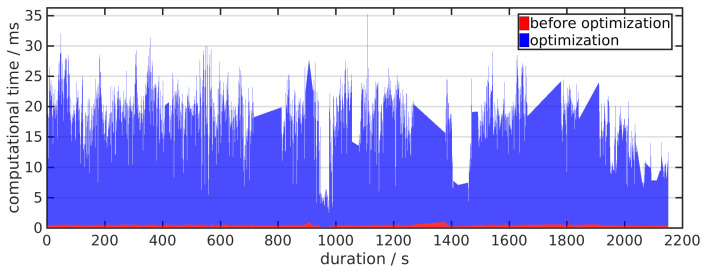
Computational time of the fusion process tested on KAIST dataset. The red component denotes the time cost on “measurement alignment” and “degeneration detection”. Although the blue component denotes the time cost on degeneration-aware batch optimization.

To validate the ability of achieving real-time localization, we evaluate the computational time cost on the multi-sensor fusion process. The computational time is defined as the duration between the time at which a new global measurement is received and the time the system state is updated by the optimization results, which is independent with the processes in the front-ends. We collect the computational time tested on the KAIST dataset and draw the results in Figure 7, in which we specifically color the the time cost on “measurement alignment” and “degeneration detection” as red. As the fusion process is taken place within a sliding-window, the computational time would not increase with time. The average processing time is 18.4 ms, which satisfies the requirement of achieving real-time global–local sensor fusion. Besides, the average time of “before optimization” is 0.45 ms, which demonstrates that our solution for the degeneration motion would not introduce large computational burden towards the fusion process.

## 5. Conclusions

To achieve drift-free 6D global localization, in this paper we propose a general optimization-based framework that fuses the 3D intermittent global positioning information and 6D local odometry for real-time global pose estimation. The system state is specifically formulated to be fully observable under general motions. To address the impact from generated motion patterns, we propose a degeneration-aware solution to robustly detect the degenerated directions that are further utilized as indicators to add prior constraints. Though extra time is cost for degeneration detection, the whole processing time could satisfy the requirement of real-time sensor fusion. The effectiveness of our proposed method is validated on both simulated and real-world dataset. In the future work, we set to extend the configuration of our framework from 3D–6D global–local combination to multiple types of pose estimators with general degree of freedom, and utilize the multi-sensor information to further detect the failure in the local pose estimators for outlier rejection.

## Figures and Tables

**Table 1:** Nomenclature.

Notation	Explanation
*C*	The sensor frame of the local estimator;
*G*	The sensor frame of the global estimator;
*O*	The reference frame of the local estimator;
*W*	The reference frame of the global estimator, which also is
	the world frame of the localization system;
*L*	The first frame in the sliding-window;
T	The transformation matrix in SE(3), where $T_{B}^{A}$ represents
	the relative transformation from frame *B* to frame *A* defi-
	ned in frame *A*;
R	The rotation matrix in SO(3), where $R_{B}^{A}$ represents the
	rotation from frame *B* to frame *A*;
q	Unit quaternion in Hamilton notation, with $q_{B}^{A}$ correspon-
	ding to $R_{B}^{A}$;
p	Translation vector in $R^{3}$, where $p_{B}^{A}$ denotes the translation
	from frame *B* to frame *A* defined in frame *A*. $p_{B_{x}}^{A},p_{B_{y}}^{A}$
	and $p_{B_{z}}^{A}$ denote the components of $p_{B}^{A}$ along x,y,z axises;
x	The system state;
s	The scale of the local estimator in $R^{+}$;
t	The timestamp of the received message;
n	The number of global measurements in the sliding-window;
$\epsilon_{a},\epsilon_{b},\epsilon_{r}$	The thresholds used for degeneration detection.

**Table 2:** Median rotational errors (deg) tested on EuRoC datasets.

	MH01	MH02	MH03	MH04	MH05
ours	1.05	0.63	1.23	1.57	0.91
full priors	1.51	1.01	1.52	4.98	3.26
no prior	1.80	0.75	2.70	2.12	1.96
VINS-Fusion	1.42	1.13	2.35	2.56	2.52
MSF	1.40	1.63	3.15	3.29	3.73

**Table 3:** ATE error (m) tested on KAIST dataset.

	GPS	Openvins	MSF	VINS-Fusion	Full Priors	No Prior	Ours
mean	5.98	14.43	21.14	9.64	9.20	/	5.58
rmse	7.15	16.46	22.09	11.11	12.21	/	6.71
std	3.91	7.91	14.47	5.52	6.40	/	3.73

**Table 4:** Median heading errors (deg) tested on KAIST dataset.

MSF	Full Priors	VINS-Fusion	Ours
12.8	1.48	1.56	1.43

**Figure A1:**
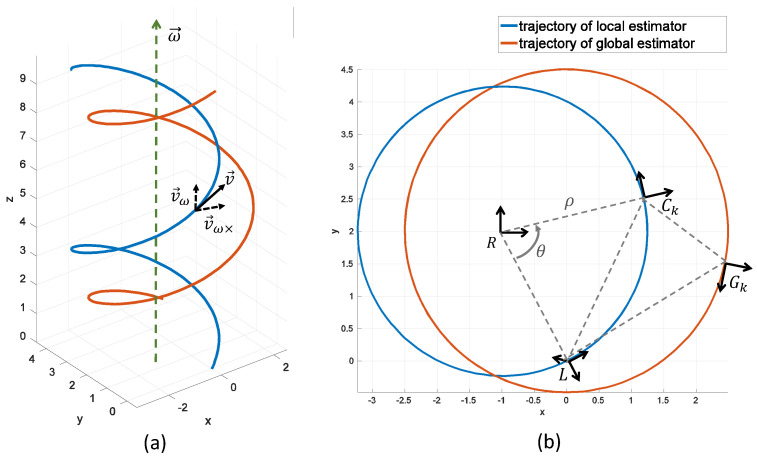
This figure demonstrates (**a**) the 3D trajectory of one-axis rotation movement with constant angular and translational velocity and (**b**) its projected trajectory viewed along the rotational axis.

## Data Availability

Not applicable.

## Abbreviations

The following abbreviations are used in this manuscript:

3/6D	3/6 Degree-of-Freedom
DC	Degeneration Constraint
EKF	Extended Kalman Filter
GPS	Global Positioning System
IMU	Inertial Measurement Unit
INS	Inertial Navigation System
LIDAR	Light Detection and Ranging
MSF	Mult-Sensor Fusion
UWB	Ultra Wide Band
VIO	Visual Inertial Odometry

## Appendix A. Derivation of the Degeneration Analysis on One-Axis Rotation with Constant Velocity

As the rotational velocity ω and translational velocity v are constant in this motion pattern, the robot can be considered as moving along a spiral curve or a circle. In the following, we denote that for a 3D vector a, it can be decomposed as $a=a_{\omega \times }+a_{\omega }$, in which $a_{\omega \times }$ represents the component that is perpendicular to ω and $a_{\omega }$ represents the component that is parallel to a. As $\left[a\times \right]\omega =\;\left[a_{\omega \times }\times \right]\omega$, we can derive $\Xi_{k}\omega$ as

(A1) $$\begin{array}{cc} \Xi_{k}\omega & =-R_{L}^{W}\left[\left(R_{C_{k}}^{L}p_{G}^{C}+sp_{C_{k}}^{L}\right)\times \right]\omega \\ \; & =-R_{L}^{W}\left[(R_{C_{k}}^{L}p_{G\omega \times }^{C}+sp_{C_{k}\omega \times }^{L})\times \right]\omega \\ \; & =-R_{L}^{W}\left[p_{G_{k}\omega \times }^{L}\times \right]\omega \end{array}$$

in which we set $p_{G_{k}\omega \times }^{L}=R_{C_{k}}^{L}p_{G\omega \times }^{C}+sp_{C_{k}\omega \times }^{L}$. We draw the projected trajectory in Figure A1b, which should be a circle with the radius ρ. As the following derivation only focus on the variables on the projected trajectory, we reuse the notations of L,C,G to represent their projected variables. For the sake of the derivation, we define a new reference frame *R*. The position of *R* is defined as the center of the projected circle, and we set its orientation the same as the reference frame *L*. As $∥p_{RC_{k}}^{R}∥\;=\;∥p_{RL}^{R}∥$, we can transform $p_{RC_{k}}^{R}$ to $R_{C_{k}}^{L}p_{RL}^{R}$. $p_{G_{k}\times }^{L}$ can be decomposed as

(A2) $$\begin{array}{cc} p_{G_{k}\omega \times }^{L} & =R_{R}^{L}\left(p_{LR}^{R}+p_{RC_{k}}^{R}+p_{C_{k}G}^{R}\right)\\ \; & =R_{R}^{L}\left(p_{LR}^{R}+R_{C_{k}}^{L}p_{RL}^{R}\right)+R_{C_{k}}^{L}p_{G}^{C} \end{array}$$

in which $p_{BD}^{A}$ denotes the vector from frame *B* to frame *D* representing in the coordinate of *A*. As $R_{R}^{L}=I_{3\times 3}$, Equation (A2) equals to

(A3) $$p_{G_{k}\omega \times }^{L}=p_{LR}^{R}+R_{C_{k}}^{L}\left(p_{RL}^{R}+p_{G}^{C}\right)$$

Thus, Equation (A1) can be derived as

(A4) $$\Xi_{k}\omega =-R_{L}^{W}\left(\left[p_{LR}^{R}\times \right]\omega +\left[R_{C_{k}}^{L}\left(p_{RL}^{R}+p_{G}^{C}\right)\times \right]\omega \right)$$

As $R_{C_{k}}^{L}$ rotates along ω, we can achieve that ${\left(R_{C_{k}}^{L}\right)}^{T}\omega =\omega$, which leads to

(A5) $$\begin{array}{cc} {}[R_{C_{k}}^{L}\left(p_{RL}^{R}+p_{G}^{C}\right)\times ]\omega & =R_{C_{k}}^{L}\left[p_{RL}^{R}+p_{G}^{C}\times \right]{\left(R_{C_{k}}^{L}\right)}^{T}\omega \\ \; & =R_{C_{k}}^{L}\left[p_{RL}^{R}+p_{G}^{C}\times \right]\omega \end{array}$$

Thus besides Equation (15), the other vector that compromises the null-space matrix is

(A6) $$N={\left[\begin{array}{cccc} \omega^{T} & {\left(R_{L}^{W}\left[p_{LR}^{R}\times \right]\omega \right)}^{T} & {\left(\left[p_{RL}^{R}+p_{G}^{C}\times \right]\omega \right)}^{T} & 0 \end{array}\right]}_{10\times 1}^{T}$$

which demonstrates that besides Equation (15), there exists one dimension of state $R_{L}^{W}$ which is along with the rotational axis ω becoming unobservable.

If the rotational and translational velocity is perpendicular, the trajectory is equal to a circle which we also demonstrate in Figure A1b. We can decompose $p_{C_{k}}^{L}$ into

(A7) $$\begin{array}{cc} p_{C_{k}}^{L} & =p_{LR}^{L}+p_{RC_{k}}^{L}=p_{LR}^{L}+p_{RC_{k}}^{R}\\ \; & =p_{LR}^{L}+R_{C_{k}}^{L}p_{RL}^{R} \end{array}$$

Thus, under this circumstance, the null-space matrix is further expanded with

(A8) $$N={\left[\begin{array}{cccc} 0_{1\times 3} & {\left(-R_{L}^{W}p_{LR}^{L}\right)}^{T} & -{\left(p_{RL}^{R}\right)}^{T} & 1 \end{array}\right]}_{10\times 1}^{T}$$

which means the scale is degenerated to be unobservable.

This completes the proof.
